# Dissemination as Dialogue: Building Trust and Sharing Research Findings Through Community Engagement

**DOI:** 10.5888/pcd13.150473

**Published:** 2016-03-17

**Authors:** Bryce McDavitt, Laura M. Bogart, Matt G. Mutchler, Glenn J. Wagner, Harold D. Green, Sean Jamar Lawrence, Kieta D. Mutepfa, Kelsey A. Nogg

**Affiliations:** Author Affiliations: Laura M. Bogart, Boston Children’s Hospital, Boston, Massachusetts, Harvard Medical School, Boston, Massachusetts, RAND Corporation, Santa Monica, California; Matt G. Mutchler, AIDS Project Los Angeles, Los Angeles, California, California State University, Dominguez Hills, Carson, California; Glenn J. Wagner, Harold D. Green, Jr, RAND Corporation, Santa Monica, California; Sean Jamar Lawrence, Kelsey A. Nogg, AIDS Project Los Angeles, Los Angeles, California; Kieta D. Mutepfa, UCLA CARE Center, Los Angeles, California. Dr McDavitt is also affiliated with University of Southern California, Los Angeles, California and Pacifica Graduate Institute, Carpinteria, California.

## Abstract

A fundamental feature of community-based participatory research (CBPR) is sharing findings with community members and engaging community partners in the dissemination process. To be truly collaborative, dissemination should involve community members in a two-way dialogue about new research findings. Yet little literature describes how to engage communities in dialogue about research findings, especially with historically marginalized communities where mistrust of researchers may exist because of past or present social injustices. Through a series of interactive community presentations on findings from a longitudinal study, we developed a process for community dissemination that involved several overlapping phases: planning, outreach, content development, interactive presentations, and follow-up. Through this process, we built on existing and new community relationships. Following each interactive presentation, the research team debriefed and reviewed notes to identify lessons learned from the process. Key themes included the importance of creating a flexible dissemination plan, tailoring presentations to each community group, establishing a point person to serve as a community liaison, and continuing dialogue with community members after the presentations. Core strategies for developing trust during dissemination included engaging community members at every step, reserving ample time for discussion during presentations, building rapport by sharing personal experiences, being receptive to and learning from criticism, and implementing input from community members. This process led to a deeper understanding of research findings and ensured that results reached community members who were invested in them.

## Introduction

Sharing research findings with community members is a vital component of community-based participatory research (CBPR) for several reasons (1,2). First, community members deserve access to the knowledge they have made possible through participation or other forms of engagement in a study (3). Second, community dissemination creates opportunities to explore the implications of research findings from a local perspective (4). Third, dissemination allows providers to implement findings immediately and locally (5), potentially reducing the gap between research and practice (6). Finally, by fostering dialogue with those most affected by a given health issue, community dissemination aids in developing culturally relevant interventions (7).

Involving community members in discussions about new findings is particularly crucial for addressing health disparities. Those who work with or are members of a target population can shed light on factors that need to be addressed (8,9); without such input interventions may be ineffective. For example, although research has identified culturally specific determinants of treatment adherence among black people living with human immunodeficiency virus (HIV) (10–13), interventions to improve adherence have rarely been tailored for this population; this lack of tailoring may account for the comparatively weak effects found in adherence intervention trials that have a substantial number of black participants (14–16). Unless health care providers and other community members are engaged in collaborative partnerships to generate insights about research findings, opportunities to render interventions responsive to culturally specific determinants may be missed.

To foster partnerships, community dissemination should involve “a two-way dialogue, not a one-way flow of information” (17). Two-way dissemination enables community interpretations of findings to be integrated as part of an iterative research process (5), and it is more likely to influence health care practice than unidirectional dissemination (6). However, the CBPR dissemination literature primarily emphasizes passive, unidirectional dissemination strategies (eg, press releases, policy briefs, newsletters, websites). Apart from brief allusions to workshops with community members (3,18,19), scant literature examines how to implement community dissemination as a two-way dialogue or address its challenges, which may include translating research terminology into lay language (20), and — when findings focus on historically marginalized communities — how to discuss research in ways that are sensitive to mistrust and concerns about being stigmatized (21–23).

This article presents lessons learned from implementing community dissemination through a series of interactive community presentations. We discuss strategies for facilitating two-way dialogue and developing trust with communities to strengthen partnerships, gain a better understanding of findings, and explore implications for culturally relevant interventions and public policy.

## Methods

**The study and the Community Advisory Board.** Project Mednet was a longitudinal study that examined how social network characteristics are associated with health outcomes and behaviors of black men and women living with HIV (24). The study was based on CBPR principles and conducted in partnership with AIDS Project Los Angeles (APLA), a large community-based organization (CBO) with an on-site co-investigator (M.G.M.) and an in-house research program with a community advisory board (CAB). We approach CBPR as a process grounded in working relationships sustained over time rather than convened for individual studies. For example, in addition to holding an academic post, the on-site co-investigator has been employed by APLA for more than 15 years, which has enabled him to develop and strengthen trusting relationships with many local providers and clients. The CAB has been sustained across multiple studies, and it comprises 6 clients and 12 service providers from 4 local social service agencies and 4 community clinics that primarily serve black people living with HIV. The service providers include HIV treatment educators, social workers, outreach staff, and HIV/AIDS clinicians. Sixteen CAB members are black, one is white, and one is Latino. For Project Mednet, CAB members provided guidance on study planning, data collection, analysis, and dissemination, including the decision to conduct a series of interactive presentations as a core community dissemination strategy. It was also important to the team and the CAB that the research team include members of the population being engaged and that staff were hired from the local community.

**The community dissemination process.** We conceptualized community dissemination as an ongoing dialogue with the community involving several overlapping phases: 1) planning, 2) outreach, 3) content development, 4) presentations with discussions, and 5) follow-up. Interactive presentations were conducted during a 6-month period, concurrent with evaluation of the process with CAB members, reinterpretation of data, and revision of presentation structure and content. Eleven presentations were conducted, with an average of 20 attendees per event. Presentation venues included 3 standing community meetings of service providers and clients, such as the Los Angeles Commission on HIV. Three were held for clients at CBOs, such as an agency that provides addiction counseling and health education to black men living with HIV. Two were held for staff at CBOs, including APLA, where most of the data were collected. One presentation was conducted as continuing education training for nurses and physicians who work primarily with black people living with HIV in South Los Angeles. Finally, 2 smaller presentations were conducted for leadership at 2 CBOs that had expressed interest in the findings: a national think tank that advocates for black people living with HIV and a grass-roots organization that provides wellness-oriented social activities for people living with HIV.

**Identifying lessons learned.** Throughout the dissemination phase, notes were collected on the interactive presentations, including content of discussions, challenges encountered, and input on findings or the dissemination process itself. During CAB meetings and in research team debriefing sessions after the presentations, preliminary themes were identified and strategies to refine the dissemination process were developed. This process continued throughout the dissemination phase, including during later small-group meetings with community providers to identify targeted strategies for implementing findings within existing programs or upcoming grant proposals.

## Planning a Community Dissemination Phase

**Build community dissemination into project aims.** A main study aim was to share findings with community stakeholders and, with their input, to identify novel intervention solutions to address medical mistrust and support adherence among black people living with HIV. Establishing this aim in the grant proposal made presentations a priority and fostered a sense of accountability among research team members. Having presentations as a formal aim also allowed us to budget funds for protected staff time to implement community dissemination. We found that funds were needed to cover staff time at multiple levels so that the research team could develop and maintain relationships with community members and CBOs. The principal investigator (L.M.B.) needed time to prepare and lead interactive presentations and engage in one-on-one communication with community members. Given her role as the research team leader, her active involvement was appreciated by community members because it demonstrated the value we placed on their input. The study coordinator (B.M.) was responsible for reaching out to CBOs, maintaining relationships, revising slide sets, coordinating logistics, conducting presentations, and facilitating discussions. A research assistant (K.A.N.) collected notes on discussions. A co-investigator (M.G.M.) also conducted interactive presentations, and an interviewer (S.J.L.) presented the study methods. Because we mainly held in-person presentations at standing meetings, expenditures for printing and space rental were minimal. However, at some presentations funds were needed for refreshments, which allowed clients and staff to attend during lunch breaks.

**Develop a plan with community members, but keep it flexible.** In collaboration with the CAB we developed an initial plan for dissemination, but because community partnerships should be flexible (25), our plan evolved iteratively in response to community feedback throughout the process. In addition to holding brainstorming sessions during CAB meetings on how to disseminate findings, we had in-depth conversations with key CAB members. We selected interactive presentations as our core dissemination strategy to create opportunities to engage community members in two-way dialogue. The research team and CAB developed a preliminary list of local venues and CBOs whose staff or clients might be interested in the findings, starting with CBOs where data had been collected or who had referred participants to us; this list evolved as new opportunities arose.

**Presenting at standing meetings is often more practical than planning special events.** Initially we considered conducting multi-hour Project Mednet public forums, but this approach was often impractical given the busy schedules of CBO staff members. CAB members suggested that instead we integrate our presentations into standing meetings, such as CBO staff meetings, continuing education trainings, or community forums where clients and staff from multiple CBOs gathered regularly. This approach resulted in strong attendance and produced in-depth discussions with a diverse range of groups, such as nurses working with people living with HIV or young gay men of color attending an HIV education program. We found that coordinators of many standing meetings were actively searching for relevant and timely content and that providing content for these meetings was a valued way of “giving back” to community members who had referred participants to us.

## Reaching Out to Community Partners

**Designate a point person to coordinate dissemination outreach.** Once the initial plan was outlined, we reached out to CBOs to explore their interest in hosting presentations. The study coordinator served as a point person for this process. Although the whole research team was active in dissemination, having a point person allowed community members to communicate with us easily, facilitated our addressing their concerns, and helped us tailor presentations to audiences. This role required experience working in the community, an ability to convey findings accurately without jargon, and readiness to answer questions about study aims, history, challenges, and community feedback.

**Draw on established relationships.** Relationships are at the heart of CBPR (1), and the dialogues initiated through interactive presentations created opportunities to strengthen our existing relationships with CBOs and community members by demonstrating that their support — through participation, referrals, or guidance — had resulted in important research findings. Ideally, the research team should have established relationships with key community gatekeepers before the dissemination phase. Several team members had long histories of engagement with the local community, and these relationships were often deepened through CAB meetings and during data collection. For example, while obtaining medical records to track patients’ engagement in care, the study coordinator developed a stronger working relationship with a local physician who treated many of the patients. During the dissemination phase, this same physician recommended our presentation as content for a continuing education series and then helped to facilitate an engaging discussion.

**Conduct “pre-meetings” with gatekeepers.** As the first step in scheduling presentations, the study coordinator initiated conversations with CBO staff members to explain our findings, to explore their possible relevance for staff members or clients, and to offer to conduct a presentation and co-facilitate a discussion. The study coordinator and CBO staff members also planned logistics and strategized together about how to ensure a good fit between content and attendees. This process began by exploring the interests and expertise of likely attendees and identifying which findings fit those interests so that slides could be tailored. The conversation also addressed how to attract attendees who would benefit from the presentation and contribute to discussions. We found that CBO staff members who had close relationships with clients were often able to identify and invite individuals or groups who cared about the research topic and about fostering a productive dialogue.

## Tailoring Content for Various Audiences

**Work with community members to select findings for presentation.** To select the overall content, we shared a broad set of findings with our CAB and asked what they thought would be of interest to community members. We then tailored this general set of findings to various audiences, such as community forums, client meetings, staff meetings, or continuing education trainings. We found that attendees at large community forums (eg, regional planning meetings where consumers and CBO representatives make recommendations on HIV-related services and funding) tended to be research-oriented and interested in precise descriptions of methods and policy implications. In contrast, meetings of CBO staff members or clients emphasized how findings could be pragmatically applied to services (eg, a one-page handout with tips for addressing clients’ mistrust). Continuing education trainings (eg, with nurses and physicians) allowed more time than other venues, enabling us to cover findings in more depth and facilitate more nuanced discussions. Continuing education attendees are regularly exposed to recent research findings at other trainings but rarely have opportunities to discuss the findings with the researchers who have conducted the studies.

## Sharing the Findings and Building Trust

**Make presentations interactive.** To foster dialogue, we included community members and CBO staff members as co-facilitators and reserved as much time for discussion as for the presentation. Whenever possible, CBO staff members helped to facilitate discussions, which led to rich dialogues combining our familiarity with the findings with their experience in the community. A project interviewer presented the methods, which strengthened rapport with attendees because he had also established relationships with CBO staff members during the study’s recruitment phase.

**Share a personal story illustrating how the issue has affected your life.** Although our research team includes several black staff members, the principal investigator and the on-site co-investigator, who often led presentations, are white. During presentations they were sometimes asked why they were doing research with black communities. In exploring these questions with the CAB, it became clear that there were concerns that nonblack researchers might be motivated by factors other than the well-being of the community, such as professional opportunism or financial gain. The team understood how such concerns could arise from both the historical and ongoing marginalization of black communities in research, health care, and other contexts. To build rapport and trust, the CAB suggested that the primary presenter address mistrust at the beginning of the presentation by telling a story that conveyed why this area of research mattered personally. When we implemented this advice, the effect in the room was palpable, establishing a feeling of personal connection between the attendees and the speaker. The story was of an experience of HIV stigma that occurred when the principal investigator, as a teenager, had requested an HIV test from her doctor, who responded in a judgmental manner, saying “We don’t have patients like that here.” She shared with the audience how that experience deepened her commitment to understanding and addressing HIV stigma through research. In addition to clarifying the speaker’s motives, sharing this story helped to humanize the research topic.

**Cultivate a receptive attitude toward criticism.** Part of seeking community feedback on research involves receiving criticism on the study’s methods, interpretation of findings, or overall approach. At such times, it may be tempting to respond defensively, for example by suggesting that the concerns are somehow less applicable to the study or researchers in question. However, we sought to adopt a receptive stance toward such comments by responding in a respectful, nonconfrontational manner. Defensive responses may undermine trust and exacerbate community concerns about researchers by dismissing the validity and relevance of their comments. We found that it was important to inquire further, seek to better understand the concerns, respond to them, learn from them, and revise our research strategies accordingly. We also came to appreciate how such comments led to deeper and more authentic conversations about issues implicit in doing research with black communities in the United States. They demonstrated community members’ investment in the research and their community — an investment that is crucial to respect and nourish in CBPR. By the end of the project, many community members confirmed that our efforts to listen to, and learn from, criticism was among the most important factors that made the study successful from a community perspective.

## Deepening Trust Through Ongoing Dialogue

**Follow up with one-on-one meetings.** After presentations, some attendees reached out to us to discuss parallels between their work as service providers and our findings. Setting up face-to-face meetings or conference calls helped to solidify new relationships and enabled us to engage in much more detailed and realistic explorations of how community partners’ work shed light on the findings, implications of the findings for their programs, and possible collaborations.

**Recognize the potential value of research findings for service providers.** Following presentations at community forums, some CBO leaders wanted to discuss how the findings related to their programs. One CBO used our preliminary data for a grant proposal, allowing for timely use of research findings (a key aim of CBPR). In another case, we presented data (from another study) to a local funder that was considering cutting funding for a community partners’ program — a program that our data suggested was yielding strong positive outcomes. For CBOs that develop new interventions, data can also suggest which aspects of their program may contribute most to improving outcomes. For example, staff members from one CBO discussed how their intervention, although developed intuitively through community input, was based on principles similar to those shown in our findings. To make findings most useful for providers, be prepared to develop brief reports or conduct tailored analyses.

**Demonstrate that suggestions are integrated into further research or programs.** In follow-up meetings, attendees and our CAB also told us that one of the most important aspects of the process was seeing that their input was implemented. Integrating feedback from presentations and follow-up conversations demonstrated to community members that the discussions were not merely academic exercises but represented mutual learning opportunities with positive effects on clients, programs, and further research.

## Discussion

We developed a dissemination process for CBPR involving a series of interactive community presentations held at local CBOs. This process was intended to deepen our understanding of our study findings, support near-term implementation by providers, strengthen relationships with community partners, and elicit ideas for culturally relevant interventions. Although researchers frequently emphasize the importance of these types of aims (4–7), few report on implementation of strategies to achieve them. Through post-presentation research team debriefings and systematic review of notes from presentations, we identified a set of concrete strategies that researchers can use to engage communities in two-way conversations about research findings. These include creating a flexible dissemination plan, tailoring presentations to various community groups, establishing a point person to serve as a community liaison, and continuing dialogue with CBOs and attendees after presentations. Keys to developing trust during dissemination included engaging community members at every step, reserving ample time for discussion during presentations, building rapport by sharing personal experiences, being receptive to and learning from criticism, and implementing community members’ input.

We found these strategies beneficial, but our findings are limited because they reflect the dissemination process of only one project. Further research is needed to determine whether the strategies can consistently foster community trust, cultural tailoring of findings, or near-term implementation by community providers. Future research could also compare different strategies. For example, as an alternative to delivering interactive presentations at CBOs, community members could be invited to day-long forums (26), which have the potential advantage of increasing one-on-one dialogue between community members and researchers. However, briefer interactive presentations may be better for reaching a wide array of community members through integration into standing meetings at CBOs, continuing education trainings, and other existing settings.

In light of growing concern that communities do not always receive the benefits of new findings (27), researchers are encouraged to design studies with community dissemination in mind (28,29). Our study illustrates how community dissemination plans that include strategies for dialogue may yield benefits beyond those limited to one-way dissemination. These strategies can be incorporated into dissemination plans and research proposals, which should specify the overall approach to be used (eg, interactive presentations) and how the approach will be implemented (eg, with a point person for community relations). Plans should also be flexible to enable responsiveness to community input and reflect the concerns of the communities being studied. For example, with populations that are historically mistrustful of research, it is important to articulate how trust has been developed or will be fostered in meaningful ways.

Conducting dissemination as a dialogue with community members involves an investment of time and resources, but it can lead to understanding of findings that is well-grounded in community perspectives and their implications for existing and new community-based services. In addition, this type of dissemination can strengthen community–academic partnerships, which builds 1) community trust in research and 2) researchers’ understanding of community concerns. New research findings may be of interest to community members, particularly if they are able to use them in proposals and programs. Given the labor-intensiveness of community dissemination, funders who support CBPR should be prepared to designate adequate resources so that community members may genuinely take an active role in all phases of research and use data for practical purposes, such as improving their programs or influencing public policy at the community level (28). For example, the National Cancer Institute developed supplemental grants for dissemination that may serve as a model for targeted dissemination; and our experience suggests it is valuable for community dissemination to be incorporated into primary funding mechanisms to encourage “designing for dissemination” (29). Similarly, academic settings should reward efforts to facilitate community dialogue on research findings, which may yield benefits not only to individual studies but also to the overall reputation of research in diverse communities.
